# A survey of Greek women's satisfaction of postnatal care

**DOI:** 10.3934/publichealth.2018.2.158

**Published:** 2018-06-12

**Authors:** Vasiliki Panagopoulou, Athina Kalokairinou, Foteini Tzavella, Styliani Tziaferi

**Affiliations:** 1 Department of Nursing, Laboratory of Integrated Health Care, University of Peloponnese, Sparta, Greece; 2 Faculty of Nursing, National & Kapodistrian University of Athens, Athens, Greece

**Keywords:** midwifery care, postnatal period, maternal satisfaction, Greece, postpartum care

## Abstract

**Background:**

The research described in this paper is a cross-sectional study which surveys women who delivered their babies in a regional hospital in Greece to investigate their satisfaction with their postnatal care. This is the first published study which measures satisfaction of postnatal services in Greece. The aim of this study is to determine which factors most influence postnatal satisfaction, which areas are lacking and therefore identify specific areas which should be targeted to improve the performance of health services.

**Methods:**

A cross sectional, quantitative study of 300 women who gave birth in a regional Greek hospital between January 2015 and July 2017 were surveyed 40 days after birth using a self-administered questionnaire. The questionnaire contained sociodemographic and clinical characteristic questions and a selection of questions from the WOMen's views of Birth Postnatal Satisfaction Questionnaire (WOMBPNSQ).

**Results:**

This study found that the dimensions with the higher satisfaction scores were “Professional support” and “Continuity”. The lower satisfaction scores were for the dimensions “Woman's health”, “Contraceptive advice” and “Social support” indicating that these are areas for improvement. The three dimensions most correlated with general satisfaction were “Time with woman”, “Feeding baby” and “Professional support”.

**Conclusions:**

This study highlights the important role of health professionals showing that they can enhance postnatal satisfaction by spending time with the women, giving guidance on the care of the newborn and baby feeding. Focusing on improving these areas is expected to enhance the quality of postnatal care.

## 1. Introduction

The postnatal period is important for the health of the mother, the newborn and the establishment of new family relationships [1]–[3]. Healthcare professionals have a key role in providing postnatal care for the mother and newborn [4],[5]. There is limited research conducted in Greece on the postnatal period, what is available mainly covers aspects of breastfeeding [6],[7] and postnatal depression [8],[9]. Until now there is no research conducted in Greece to assess the effectiveness of the provided postnatal care and the parents' satisfaction from the care they receive postnatally. This study aims to fill this research gap. The United Nation's Global Strategy for Women's, Children's and Adolescents' Health 2016–2030 10 points out the importance of providing quality care in all settings for new mothers and new babies. In light of this strategy postnatal maternity health services in Greece should be assessed and changes should be implemented for the benefit of the new mother, the new baby and the new family.

Sadly, postnatal care is frequently not given as much attention as pregnancy and labour [11]–[14]. During the postnatal period some women express dissatisfaction from the health care services provided [15]–[18]. Brown et al. 15, in Australia, identified that after labour the new mothers felt that the midwives did not show enough sensitivity to their needs as they seemed to be always in a hurry and did not spend enough time with them. When the women got discharged home from hospital they felt that they did not have enough information and knowledge for how to best look after their baby. Rudman and Waldenström 16 investigated women's negative views of the postnatal care they received in a hospital in Sweden. The patients reported difficulty in receiving personalised care and help with breastfeeding. Razurel et al. 17 found that the women in Switzerland felt that the education they received during pregnancy did not help them when problems and concerns arouse in the postnatal period. The women expressed their need to have further help and support during the postnatal period from health care professionals. Another study from Vancouver, Canada 18, found that the time the women spent in the postnatal ward did not prepare them adequately for the first weeks with their baby at home. A study in Turkey concluded that new mothers were not sufficiently prepared for the postpartum period 19.

Whilst there have been studies of women's experiences of pregnancy, labour and childbirth in Greece [20]–[22] there are no studies covering women's experiences and satisfaction of their postnatal care. Postnatal care in Greece is provided mainly in the hospital. The new mother stays in the hospital in average for four days after the birth of her baby and then she is discharged home. The new mother returns to the hospital at around 40 days after her discharge for the doctor to check her recovery from childbirth. The research described in this paper investigates the satisfaction of the women who delivered in a regional hospital in Greece with their postnatal care to help fill the gap in this area of research and identify areas for improvement in clinical practice. Patient satisfaction is considered to be an important indicator for health care quality and at the same time is a significant quality improvement tool for health care providers [23],[24].

## 2. Materials and methods

### 2.1. Study setting, participants & selection

A total of 300 women who gave birth in a regional Greek public hospital between January 2015 and July 2017 were surveyed 40 days after the birth of their baby to assess their satisfaction with the postnatal care they received. Inclusion criteria were women aged over 18, who delivered a healthy term infant. Mothers were informed that no personally identifying information would be recorded at any point of the survey and verbal informed consent was given by the women before they voluntarily completed the self-administered questionnaire. 375 women were approached during the two and a half year research period and asked to take part to the research, 25 women declined (7%) to participate. Another 50 women did not meet the inclusion criteria (13%) so were also excluded from the study leaving a total of 300 women surveyed; the survey was stopped after 300 completed responses had been received. Excluded women were those under 18 (30 women, 6%), women who had not delivered a healthy term infant (7 women, 2%), or were illiterate and could not complete the questionnaire (13 women, 4%). During the period that study was conducted a total of 850 deliveries were performed in the Greek regional hospital the research took place. The final sample of 300 women was obtained by randomly selecting the post partum women and was considered to be a representative sample of the population of postnatal women of the hospital where the study took place.

### 2.2. Questionnaire

Ethical approval for this research was granted from the scientific committee of the regional Greek public general hospital (18^th^ Dec 2014, approval number Φ/Γ/2/14962). New mothers completed the questionnaires in the postnatal ward before attending their 40 day postnatal check after birth. The questionnaires included questions, translated to Greek, from the WOMen's views of Birth Postnatal Satisfaction Questionnaire (WOMBPNSQ), which is a psychometric multidimensional postnatal satisfaction questionnaire 25. The questions used in this study from the WOMBPNSQ questionnaire were translated from English to Greek and back translated from Greek to English by two language experts. Questions not used in this study from the WOMBPNSQ questionnaire 25 where those relating to the dimensions “Postnatal visiting”, “Health visitor care” and “GP care” as these services are not usually provided and these professionals are not generally involved in the provision of postnatal care in the Greek National Health Service. Additional questions were added so that correlations with the demographics and the obstetric history of the women could be identified; a list of these questions is given in Tables 1 and 2.

**Table 1.** Demographics of the women surveyed.

Number of people surveyed	300	
Maternal age, mean (SD)	31.3	(5.4)
Marital status	No.	(%)
Married	272	(90.7%)
Not Married	28	(9.3%)
Educational level	No.	(%)
Primary	28	(9.3%)
High School	40	(13.3%)
Senior High	98	(32.7%)
College Certificate	43	(14.3%)
Technological University Degree	41	(13.7%)
University Degree	38	(12.7%)
Postgraduate Degree	12	(4.0%)
Employment		
Working, No. (%)	109	(36.5%)
Not Working, No. (%)	190	(63.5%)
If working how many weeks leave do you have? mean (SD)	7.0	(4.4)
How many children do you have including the newborn?	No.	(%)
1	117	(39.0%)
2	84	(28.0%)
3	37	(12.3%)
> 3	9	(3.0%)

### 2.3. Data analysis

Α pilot study, with a sample of 80 new mothers conducted prior to the survey, checked that the questions were understood by the women and verified that the internal reliability of the satisfaction questions was good or acceptable (Cronbach's Alpha typically above 0.6 and maximum 0.82) 26.

A seven point Likert scale was used for the satisfaction questions. Mean values and Standard Deviations (SD) were used for the description of the quantitative variables. Absolute (N) and relative (%) frequencies were used to describe the dichotomous variables. Student's t-test was used to compare two groups of quantitative variables. The Pearson correlation coefficient (r) was used to measure the correlation between two quantitative variables. The significance of this correlation was checked by calculating the probability “*p*” that this correlation occurred by chance (the null hypothesis), a *p* value less than 0.05 was considered to be statistically significant. The internal reliability of the questionnaire was tested using Cronbach's Alpha.

The cross-correlations between the dimensions were investigated by calculating the correlation coefficient between each dimension. The *p*-value probability that this correlation occurred by random variability was also calculated.

**Table 2.** Prenatal statistics of the women surveyed.

In general, would you say your health is:	No.	(%)
Very good	178	(61.0%)
Good	108	(37.0%)
Moderate	5	(1.7%)
Poor	0	(0.0%)
Very poor	1	(0.3%)
Childbirth Type	No.	(%)
Natural	157	(52.3%)
Forceps	22	(7.3%)
Emergency Caesarean	44	(14.7%)
Planned Caesarean	76	(25.3%)
Induced Labour?	No.	(%)
Yes	94	(32.3%)
No	197	(67.7%)
Epidural anesthesia?	No.	(%)
Yes	78	(27.4%)
No	207	(72.6%)
Do you breastfeed?	No.	(%)
Yes	269	(90.6%)
No	28	(9.4%)
If yes, do you breastfeeding exclusively?	No.	(%)
Yes	103	(39.9%)
No	155	(60.1%)
If not, duration in days of breastfeeding?	Mean	(SD)
	15	(19.1)
How many babies born in this birth?	No.	(%)
1	291	(97.0%)
2	7	(2.3%)
Did the baby need to be hospitalized in the neonatal unit?	No.	(%)
Yes	13	(4.3%)
No	286	(95.7%)
	Mean	(SD)
How many days did you stay in the hospital after childbirth?	3.7	(0.7)
	Mean	(SD)
Gestational age, in weeks, at delivery	38.3	(2.4)
	Mean	(SD)
Infant birth weight (g)	3207	(465.5)
Were there complications in pregnancy?	No.	(%)
Yes	11	(3.7%)
No	289	(96.3%)
Were there complications in childbirth?	No.	(%)
Yes	14	(4.7%)
No	286	(95.3%)

## 3. Results

The 300 women surveyed had a mean age of 31 and a wide range of educational levels, Table 1. Thirty six percent of the women were employed and most (91%) were married. It is common practice to induce women in Greece which explains the relatively high proportion of induced labours (32%). The rate of epidural or spinal anaesthesia was 27%. Of childbirths 53% were natural and the remainder either by forceps (7%), planned (25%) or unplanned caesarean (15%), Table 2.

A summary of the main results of the questionnaire is given in Table 3. The results of the questions were grouped into the same satisfaction dimensions used in the original WOMBPNSQ questionnaire 25. The internal reliability of the dimensions was generally good, having internal reliability with Cronbach's Alpha over 0.6, with maximum 0.858, Table 4. The “Feeding baby” and “Social support” dimensions had less than ideal reliability with Alpha of 0.63 but were retained as these were used in the original WOMBPNSQ study. The dimensions with the higher internal reliability were “Professional support”, “Partner support” and “Time with the woman”, which all had coefficients above 0.8. The dimensions with the higher mean scores were “Professional support” and “Continuity” which indicate a higher satisfaction in these areas. The dimensions “Woman's health”, “Contraceptive advice” and “Social support” had the lower satisfaction scores indicating that these areas could be improved.

It was found that many of the dimensions are cross-correlated with each other and the general satisfaction scale; these cross-correlations are statistically significant as they are unlikely to have occurred by chance (*p* < 0.001), Table 5. The three dimensions most correlated with general satisfaction were “Time with woman”, “Feeding baby” and “Professional support”.

**Table 3.** Results of the questionnaire.

Question	Mean score	Standard deviation
My partner/husband could not have supported me any better in any possible way	79%	23%
My carers explored adequately with me my contraceptive needs	65%	21%
For my postnatal care I always saw the same carer(s)	67%	24%
My carers often appeared rushed	27%	18%
I needed to be at home much sooner after the birth	42%	25%
There are things about the postnatal care system where I received my care that need to be improved	53%	23%
Sometimes carers made me feel a little foolish	20%	17%
The amount of time that I spent in hospital after my baby was born was about right	74%	17%
I would have liked more advice on feeding my baby	38%	24%
My partner/husband was the best possible help to me after the baby was born	68%	26%
Carers never acted too businesslike and impersonally towards me	80%	17%
Carers usually spent plenty of time with me	72%	18%
I was given little advice on contraception following the birth of my baby	42%	22%
I was in a fair bit of pain in the first few days/weeks after the birth	53%	28%
My postnatal care went nearly exactly as I had hoped it would	76%	16%
Many different carers provided me with postnatal check ups	59%	24%
I was given an excellent explanation of why I experienced after-pains and how I could cope with them	69%	17%
My postnatal care just seemed to involve a series of routine procedures	48%	19%
I made new friends during the days/weeks after the birth of my baby	56%	21%
It would have been so much better if I had had a longer hospital stay after the birth	27%	18%
I didn't need a lot of pain relief after the birth	55%	28%
My carers acted professionally at all times	78%	17%
The postnatal care that I received was just about perfect	77%	17%
Meeting in the postnatal days/weeks other women who had recently given birth was of no use to me	39%	18%
Those who provided my postnatal care sometimes hurried too much when they treated me	29%	19%
I could have had just a very little more help from my birth partner/husband	31%	24%
A little more time being spent on my health would have been welcome	52%	23%
I needed more time in hospital to get used to caring for my new baby	26%	18%
My carers rarely left me feeling that I didn't know what was best for my baby	73%	18%
It was reassuring to meet other women like me after my baby was born	66%	17%
My carers were never insensitive nor lacked understanding	77%	19%
I would have liked more chance to talk to my carers for medical advice about care of myself	46%	23%
I was given lots of help on how to feed my baby	75%	19%
My carers discussed the full range of contraception options with me following the birth of my baby	59%	22%
The carers who treated me should sometimes have given me just a little more respect	24%	17%
I needed more time with my carers so that they could help me more	35%	23%
There are some things about the postnatal care that I received that could have been better	47%	24%
After the birth I would have liked more chance to talk to doctors for medical advice	40%	23%
All my carers always treated me in the most friendly and courteous manner possible	80%	16%
My partner met all my needs after the birth	77%	20%
After the birth, carers always had lots of time to discuss problems with me	69%	19%
I could have done with more time for my body to adjust after the birth before going home	31%	19%
Sometimes carers did what was easier for them and not what seemed best for me	24%	19%

**Table 4.** Satisfaction dimensions and comparison with UK survey.

Dimension	Survey in Greece (these results)			Survey in UK (from Ref 25)			t-test *p* value (that Mean of Greece-UK samples different by chance)
	Mean	SD	Cronbach's Alpha	Mean	SD	Cronbach's Alpha	
General satisfaction	68.6	16.0	0.764	41.8	21.8	0.848	< 0.001
Inpatient stay	72.2	15.4	0.781	31.0	21.6	0.861	< 0.001
Woman's health	53.9	18.9	0.757	37.3	19.8	0.825	< 0.001
Contraceptive advice	60.6	17.5	0.746	40.5	23.8	0.855	< 0.001
Feeding baby	71.6	17.9	0.629	41.7	13.6	0.778	< 0.001
Partner support	71.3	20.0	0.821	24.5	21.6	0.839	< 0.001
Social support	61.0	14.1	0.637	49.9	16.6	0.744	< 0.001
Professional support	78.1	13.8	0.858	27.5	18.7	0.744	< 0.001
Pain after birth	73.1	24.7	0.731	53.9	27.8	0.779	< 0.001
Time with woman*	70.1	15.6	0.817	N/A*	N/A*	N/A*	N/A*
Continuity	74.2	20.9	0.766	59.4	23.4	0.735	< 0.001

*Dimension not included in WOMBPNQ4 so UK results not published in Ref 25, this survey uses the full set of questions and dimensions from WOMBPNQ3.

**Table 5.** Cross-correlations of the Satisfaction Dimensions with *p*-value probabilities given below in (brackets).

Dimension	General satisfaction	Inpatient stay	Woman's health	Contraceptive advice	Feeding baby	Partner support	Social support	Professional support	Pain after birth	Time with woman	Continuity
General satisfaction	1.000	0.284	0.588	0.438	0.692	0.344	0.395	0.698	0.191	0.733	0.077
	N/A	(< 0.001)	(< 0.001)	(< 0.001)	(< 0.001)	(< 0.001)	(< 0.001)	(< 0.001)	(< 0.001)	(< 0.001)	(0.456)
Inpatient stay	0.284	1.000	0.422	0.102	0.504	0.150	0.170	0.333	0.297	0.305	0.302
	(< 0.001)	N/A	(< 0.001)	(0.077)	(< 0.001)	(0.009)	(0.003)	(< 0.001)	(< 0.001)	(< 0.001)	(0.003)
Woman's health	0.588	0.422	1.000	0.444	0.714	0.260	0.141	0.495	0.185	0.583	0.068
	(< 0.001)	(< 0.001)	N/A	(< 0.001)	(< 0.001)	(< 0.001)	(0.015)	(< 0.001)	(0.001)	(< 0.001)	(0.513)
Contraceptive advice	0.438	0.102	0.444	1.000	0.360	0.158	0.266	0.333	0.037	0.460	0.055
	(< 0.001)	(0.077)	(< 0.001)	N/A	(< 0.001)	(0.006)	(< 0.001)	(< 0.001)	(0.523)	(< 0.001)	(0.595)
Feeding baby	0.692	0.504	0.714	0.360	1.000	0.044	0.248	0.699	0.190	0.365	0.255
	(< 0.001)	(< 0.001)	(< 0.001)	(< 0.001)	N/A	(0.662)	(0.012)	(< 0.001)	(0.057)	(< 0.001)	(0.012)
Partner support	0.344	0.150	0.260	0.158	0.044	1.000	0.251	0.348	0.039	0.365	0.102
	(< 0.001)	(0.009)	(< 0.001)	(0.006)	(0.662)	N/A	(< 0.001)	(< 0.001)	(0.503)	(< 0.001)	(0.321)
Social support	0.395	0.170	0.141	0.266	0.248	0.251	1.000	0.409	0.123	0.426	0.055
	(< 0.001)	(0.003)	(0.015)	(< 0.001)	(0.012)	(< 0.001)	N/A	(< 0.001)	(0.033)	(< 0.001)	(0.594)
Professional support	0.698	0.333	0.495	0.333	0.699	0.348	0.409	1.000	0.257	0.746	0.270
	(< 0.001)	(< 0.001)	(< 0.001)	(< 0.001)	(< 0.001)	(< 0.001)	(< 0.001)	N/A	(< 0.001)	(< 0.001)	(0.008)
Pain after birth	0.191	0.297	0.185	0.037	0.190	0.039	0.123	0.257	1.000	0.237	0.153
	(< 0.001)	(< 0.001)	(0.001)	(0.523)	(0.057)	(0.503)	(0.033)	(< 0.001)	N/A	(< 0.001)	(0.136)
Time with woman	0.733	0.305	0.583	0.460	0.691	0.365	0.426	0.746	0.237	1.000	0.087
	(< 0.001)	(< 0.001)	(< 0.001)	(< 0.001)	(< 0.001)	(< 0.001)	(< 0.001)	(< 0.001)	(< 0.001)	N/A	(0.401)
Continuity	0.077	0.302	0.068	0.055	0.255	0.102	0.055	0.270	0.153	0.087	1.000
	(0.456)	(0.003)	(0.513)	(0.595)	(0.012)	(0.321)	(0.594)	(0.008)	(0.136)	(0.401)	N/A

No significant correlation was found between any of the satisfaction dimensions and Maternal age, Number of children or Infant birth weight. Education level was found to be statistically significantly correlated (*p*-value less than 0.0001) with the Partner support dimension with a correlation coefficient of 0.31; this shows that more highly educated women are, generally, more satisfied with the support given to them by their partners than less well educated women. The mean satisfaction value of women who work *vs.* the women who don't work was only statistically significantly different (t-test *p*-value greater than 0.05) for the “Partner support” dimension (mean value 75.6% for working women *vs.* 69.1% for non-working women, t-test *p*-value = 0.006). Working women were also found to be older (mean age of working women 32.8, *vs.* 30.4 not working, t-test *p* = 0.0001) and more highly educated e.g. more likely to have a university degree or higher (t-test *p* < 0.0001). Age was correlated with education level with a correlation coefficient of 0.12 (t-test *p* = 0.047). The findings of this study are that younger women are more likely to breastfeed (mean age of women who breastfed 31.5 *vs.* 33.5 mean age of women who did not, t-test *p* that these mean values are different by chance = 0.021). Women who breastfed were also positively correlated with those who gave a higher score on the “Feeding baby” satisfaction dimension, mean score 73.2 for those who breastfed *vs.* 45.8 for those who didn't (t-test *p* = 0.002). Breast feeding women also had a higher general satisfaction compared to those who didn't (mean 69.4% *vs.* 60.9%, t-test *p* = 0.007), higher satisfaction with professional support (mean 79.1% *vs.* 70.1%, t-test *p* = 0.001) and higher satisfaction on the “Time with woman” dimension (71.1% *vs.* 60.4%, t-test *p* = 0.0006).

The fact that women who have not previously given birth are more likely to be induced in Greece is also seen in the statistical analysis. Women who have been induced have a statistically significant (t-test *p*-value less than 0.01) lower mean number of children than those that have not been induced (1.2 *vs.* 1.6 respectively).

## 4. Discussion

The dimensions with the higher mean scores were “Professional support” and “Continuity” which indicate a higher satisfaction for new mothers in these areas. “Professional support” and “Time with woman” were found to be the dimensions most strongly correlated with the general satisfaction dimension. These results are in agreement with the results of other studies which found that improved professional support, including continuity of midwifery care, results in higher satisfaction rates of postpartum women [27],[28]. This is particularly relevant as multiple studies [29]–[32] have found that there is frequently a lack of continuity or absence of adequate care in the postpartum period.

The dimensions with the lower mean satisfaction scores were “Woman's health”, “Contraceptive advice” and “Social support” which indicate lower satisfaction of new mothers in these areas. These results show that postnatal women need more time from health care professionals to get professional advice about their health and wellbeing. Health care providers should also empower women to mobilise social support that will help them in their parenting role and psychological health. Recent studies [33]–[35] have shown the important role of health professionals in maternal health promotion and wellness. The findings of this study show that, if health care professionals improve these areas on clinical practice, a better quality postnatal care will be provided to postnatal women to improve new mothers satisfaction on these areas of their postnatal care.

Using the same dimensions as the original study enables direct comparison between the women surveyed in this research and those surveyed by Smith 25 in the UK. What is clear from the results is that the women in this study have responded differently to those from the original study, Table 1. This difference is tested statistically and it is found that t-test *p* values are all less than 0.001 indicating that it is unlikely that the different mean values of satisfaction between the two studies appear because of random variability in the results. However, the values of the internal reliability, as measured by Cronbach's Alpha, remain within acceptable limits so whilst the level of the women's satisfaction in each of the dimensions is different from those in the UK, the dimensions themselves remain a valid measurement tool. In general, the women surveyed in this research report higher levels of satisfaction than those in the UK resulting in higher mean values in all of the dimensions. The replies from the different women in this study also showed lower variability than those in the UK, resulting in a lower standard deviation.

There are many possible reasons for the differences in the results of the two studies, like differences in the hospital protocols and policies, differences in the method of collecting the survey results, differences in the level of women's expectations and differences in the settings of the studies.

The policy and protocols of the hospital in this study accommodate the family support by allowing the new mother to have one or two people with her 24 hours a day during her hospital stay. It is common for the mother or mother-in-law to stay with the woman during her postnatal stay to assist with the care of the baby. The visiting times as well as the number of visitors are also flexible, so extended family and friends are allowed to visit as and when they like with the agreement of the new mother. On the contrary, this is not the case in the UK. The new mother is not usually allowed to have someone with her 24 hours a day. Even her partner is allowed to visit during the visiting hours. There are typically restrictions on the number of visitors and the new mother is allowed to have only two visitors by her bedside at a time. The flexible visiting times of the new mother may contribute to the increased satisfaction levels in the local Greek hospital in this research compared with UK hospitals.

Whilst the questions asked in the survey were the same with the study by Smith 25, translated from English to Greek and back translated from Greek to English, there are differences in the method used to collect the survey replies from the women. Smith 25 sent the questionnaires by post to women who completed the forms and then also returned them by post. In this survey women completed the questionnaires at the hospital. It is not known at this stage how much, if any, this changed the results; a future study could be completed using the postal method for women in Greece. However, it is noted that the Greek postal system is less customer focused than the UK postal system thus women in Greece would be less likely to return the survey. Women in the UK simply need to put the reply questionnaire in the prepaid envelope and post it in one of the many post boxes, whilst women in Greece may have a lengthy wait in the post office to post the envelope with the questionnaire.

The hospitals in Greece have significant shortages of staff, equipment and some materials/medication due to the financial crisis [36],[37]. With the lack of staff and equipment it might have been expected that women's satisfaction of their care would be lower in Greece than the UK but this was not found in this study. Perhaps, the fact that the women are aware of the difficult circumstances, and they are also affected by the financial crisis, lowers their expectations which are then exceeded by the hard work of the staff [38],[39] resulting in higher levels of satisfaction.

The present study in Greece took place at a regional general hospital. The maternity clinic in this hospital includes the delivery suite and the antenatal and postnatal wards on the same floor. The staff working in the clinic is involved with the care of the women from the time they are admitted to the ward in labour through the delivery and their postnatal stay. This gives the women continuity of care as the same health professionals are involved at all stages of their care. However, the UK study covered a variety of maternity services including different care models in different settings. This could have a direct effect on the results produced for the UK study.

The single location used in this research is a limitation of this study and is likely to be at least part of the reason for the lower standard deviation of the results compared to the UK study. We also note that the setting of this study is different from Urban Greek maternity hospitals. For example, in Athens the maternity hospitals have separate labour wards, antenatal wards and postnatal wards. The new mothers see different healthcare professionals during different stages of their care. A recommendation for future research is to measure maternal satisfaction in the large maternity hospitals in metropolitan areas of Greece. This would enable a comparison of the results of this study with the satisfaction of new mothers in larger population areas of Greece.

The findings of this study in a regional Greek Hospital show that the satisfaction levels of new mothers from the postnatal care they receive are good. However, it is also clear that postnatal women need more from their carers, in respect of their general health and wellbeing. The traditional health care model in Greece deals with targeting health problems. Postnatal women need advice on health promotion, contraception and transition to motherhood. The findings of this research have implications for clinical practice as changes should be implemented. Health care professionals should assume their role in health promotion empowering women to acquire the right skills for the postnatal period.

## 5. Conclusions

This study surveyed 300 women in a regional hospital in Greece to determine their satisfaction of the postnatal care they received. The findings of this research show that health professionals can play a key role in enhancing satisfaction of new mothers with their postnatal care by addressing their needs and expectations. It is recommended that health care professionals spend quality time with the postnatal women giving them patient centred care and advice on mother's health, baby care and feeding. There is certainly place for improvement in clinical practice, as the postnatal care provided is not ideal and yet there is a clear need for a more holistic and personalised care. Health care providers should offer holistic care and try not only to deal with problems, but also to promote maternal health and well being to educate new mothers for the parental skills they need to care for their baby and empower women to mobilise social support. These recommendations for changes in clinical practice are expected to improve the satisfaction of the women and at the same time would improve the health and well being of the new parents and the neonate.
